# Adipokines as Drug Targets in Diabetes and Underlying Disturbances

**DOI:** 10.1155/2015/681612

**Published:** 2015-04-08

**Authors:** Vinícius Andrade-Oliveira, Niels O. S. Câmara, Pedro M. Moraes-Vieira

**Affiliations:** ^1^Laboratory of Transplantation Immunobiology, Department of Immunology, Institute of Biomedical Sciences IV, University of São Paulo, SP, Brazil; ^2^Laboratory of Clinical and Experimental Immunology, Nephrology Division, Federal University of São Paulo, SP, Brazil; ^3^Division of Endocrinology, Diabetes, and Metabolism, Department of Medicine, Beth Israel Deaconess Medical Center, Harvard Medical School, MA, USA

## Abstract

Diabetes and obesity are worldwide health problems. White fat dynamically participates in hormonal and inflammatory regulation. White adipose tissue is recognized as a multifactorial organ that secretes several adipose-derived factors that have been collectively termed “adipokines.” Adipokines are pleiotropic molecules that gather factors such as leptin, adiponectin, visfatin, apelin, vaspin, hepcidin, RBP4, and inflammatory cytokines, including TNF and IL-1*β*, among others. Multiple roles in metabolic and inflammatory responses have been assigned to these molecules. Several adipokines contribute to the self-styled “low-grade inflammatory state” of obese and insulin-resistant subjects, inducing the accumulation of metabolic anomalies within these individuals, including autoimmune and inflammatory diseases. Thus, adipokines are an interesting drug target to treat autoimmune diseases, obesity, insulin resistance, and adipose tissue inflammation. The aim of this review is to present an overview of the roles of adipokines in different immune and nonimmune cells, which will contribute to diabetes as well as to adipose tissue inflammation and insulin resistance development. We describe how adipokines regulate inflammation in these diseases and their therapeutic implications. We also survey current attempts to exploit adipokines for clinical applications, which hold potential as novel approaches to drug development in several immune-mediated diseases.

## 1. Introduction

Diabetes and obesity are serious health threats and increase the risk for many chronic diseases. The World Health Organization predicts that 3 billion adults will be either overweight or obese by 2015 [1]. Obesity is one of the strongest risk factors for the development of type 2 diabetes [2]. A major cause of type 2 diabetes is impaired insulin action in adipose tissue (AT), skeletal muscle, and the liver coupled with insufficient secretion of insulin to overcome this resistance. Even without diabetes, insulin resistance is a major risk factor for cardiovascular disease and early mortality [3, 4]. To develop new strategies for the prevention and/or treatment of obesity-related diseases, an improved understanding of the cellular and molecular mechanisms underlying obesity and their cross talk with other systems, such as the immune system, is essential.

**Figure 1:**
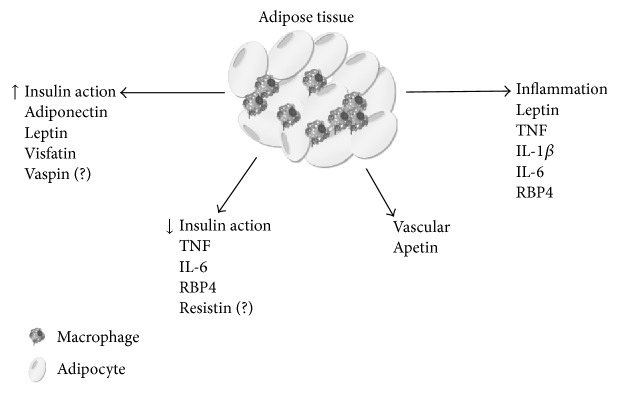
The adipose tissue secretes molecules called adipokines. These adipokines act locally (adipose tissue) and systemically, regulating several aspects of body homeostasis, including the immune system, the cardiovascular system, and insulin sensitivity.

Obesity leads to a dysregulation of several immunologic and adipose tissue-derived factors. Adipose tissue secretes many adipokines, including RBP4 (retinol binding protein 4), leptin, resistin, vaspin, visfatin, hepcidin, adiponectin, and inflammatory cytokines, which act locally (autocrine/paracrine) and systemically (endocrine) to regulate insulin sensitivity, immune response, cardiovascular function, and many physiological processes [5] (Figure 1). Adipocyte-secreted molecules can either enhance (e.g., adiponectin) or impair (e.g., TNF, interleukin-*β* (IL-1*β*), and resistin) insulin action [4, 6]. An effective approach for the treatment of obesity and its related disorders would involve a systematic investigation of factors affecting energy intake, systemic low-grade inflammation, adipose and liver inflammation, metabolism, and energy expenditure. At the individual level, interventions to treat overweight have revealed limited success with the exception of bariatric surgery, which has a relatively high mortality risk. Adipokines play an important role in the regulation of appetite and satiety, inflammation, fat distribution, insulin sensitivity, and energy expenditure, among others, and are promising molecular candidates for the development of new treatments for obesity and its related diseases.

## 2. Leptin

**Figure 2:**
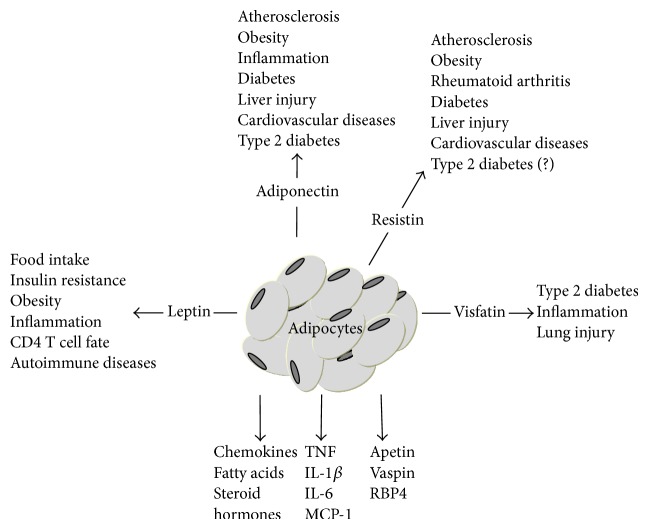
The adipose tissue secretes several hormone-like molecules called adipokines. Adipokines are pleiotropic molecules, participating in body physiology regulation. Adipokines also play a role in several diseases such as diabetes, atherosclerosis, and autoimmune diseases, among others.

Leptin acts as an endogenous sensing factor and provides a critical link between the environment, metabolism, and immune function [4, 6, 7] (Figure 2). Leptin was first described in 1994 [8], although insights into the biology of an unknown regulator of body weight and food intake were gained earlier with the description of two mouse models, one obese (ob) and the other diabetic (db) [9, 10]. Leptin signals via the leptin receptor (ObR), which exists as several isoforms; these receptors have identical extracellular domains, but only ObRb has a long cytoplasmic domain and definitive signaling capacity through the Janus Kinase (Jak) and STAT3 and also AkT/mTOR pathway [11].

Leptin is an important regulator of the immune system. Leptin-deficient (ob/ob) and leptin receptor-deficient (db/db) mice exhibit thymic atrophy and are immunodeficient [12]. Leptin is considered a proinflammatory adipokine because of the actions it exerts in several cells of the immune system, including monocytes/macrophages, dendritic cells, neutrophils, eosinophils, basophils, natural killer cells, and lymphocytes [4, 6, 13, 14]. In humans, leptin was shown to shift CD4 T cells toward a Th1 profile and to stimulate both the activation and the proliferation of circulating monocytes.

In the innate immune system, leptin participates in the activation of monocytes and macrophages, supporting phagocytosis and the production of leukotriene, cyclooxygenases, nitric oxide, IL-18, and proinflammatory cytokines TNF and IL-1 [15–17]. Also, leptin stimulates both the activation and the proliferation of circulating human monocytes [18]. In mice prone to lupus development, leptin increases macrophages phagocytosis of apoptotic cells by increasing cAMP levels, favoring the availability of self-antigen from apoptotic cells [19]. In obese and insulin-resistant mice, leptin levels increase due to leptin resistance. These high circulating levels of leptin can act on adipose tissue macrophages, increasing the levels of proinflammatory M1 macrophages [20]. In neutrophils, leptin increases chemotaxis and the release of oxygen radicals indirectly via the production of TNF by macrophages [21, 22]. Leptin affects the activation and development of NK cells, both in vivo and in vitro [23, 24]. Because NK cells express OBRb and db/db mice exhibit an NK cell deficit, leptin may act both on the development and maintenance of these cells [23]. Dendritic cells, key cells in antigen presentation and induction of adaptive immune system activation [25], are also modulated by leptin. Leptin promotes the maturation and survival of DCs by activating nuclear factor-*κ*B (NF*κ*B), which exerts an antiapoptotic effect on DCs [26]. Treatment of monocyte-derived DCs with leptin increases the production of IL-12, IL-6, and IL-1*β* and downregulates the production of IL-10, underlining the view of leptin as a proinflammatory adipokine [26]. Furthermore, both immature (iDC) and mature (mDC) bone marrow-derived DCs (BMDCs) from ob/ob and db/db mice demonstrated decreased expression of costimulatory molecules and an impaired ability to stimulate allogeneic [27] or syngeneic [13] T cell proliferation. Importantly, leptin blocks the capacity of DCs to induce regulatory T cells [13]. Although several reports describe the role of leptin in dendritic cells, how leptin affects tissue resident dendritic cells remains unclear. One study analyzing splenic resident dendritic cells reported that splenic DC from leptin-deficient mice can enhance T cell response ex vivo [28], a result that contrasts those obtained with bone marrow-derived dendritic cells. Thus, it seems that leptin may have different role according to DC subset. Thus, strategies to reduce the levels of circulating leptin could be important for inducing a regulatory type of immune response.

In adaptive immunity, leptin has been primarily investigated in CD4^+^ T cells. Lord and coworkers showed that leptin exerts a specific effect on T cell responses by regulating the proliferative responses of naïve and memory T cells and increasing IFN-*γ* production [29]. Followed by this study, leptin was shown to enhance the proliferation of human T cells and shift the CD4 T cell profile towards a Th1 phenotype [30]. Recently, a role for leptin in determining CD4^+^ T cell fate has been described. Leptin favors both inflammatory Th1 and Th17 cells [26, 29] and inhibits both Th2 and regulatory T cells (Treg) [13, 31, 32]. Leptin-deficient mice exhibit increased Treg numbers and enhanced Treg suppressive potential [33, 34]. Furthermore, blockade of leptin receptors during TCR activation induces Treg proliferation [32]. T cells from ob/ob animals exhibit decreased secretion of proinflammatory cytokines, such as IL-2, IFN-*γ*, TNF, and IL-18, and increased secretion of Th2 cytokines, such as IL-4 and IL-10, after mitogenic stimulation, which can explain why these animals demonstrate greater protection from autoimmune diseases and transplantation rejection [34–36]. Similarly, leptin receptor-deficient mice (db/db) have impaired IFN-*γ* production by T cells as well as T cell proliferation and Th17 differentiation [37]. Leptin increases Th1 responses and inhibits Th2 immune responses, affecting the polarization of CD4^+^ T cells towards a Th1 phenotype [29]. Using conditional deletion for LepR in CD4 cells, Saucillo and coworkers showed that leptin signaling pathway is associated with upregulation for the glucose transporter 1 in CD4^+^ T cells [37], which is an important receptor for glucose uptake and glycolysis in CD4 T cells, helping their activation and effector function [38]. Because leptin favors the Th1 and Th17 profile and these cells have been associated with autoimmune diseases, such as experimental autoimmune encephalomyelitis (EAE), it is possible that leptin neutralization can, at least partially, protect against the development of transplant rejection, EAE, and other autoimmune diseases, such as type 1 diabetes, lupus, and antigen-induced arthritis [13, 33, 34, 39, 40]. Indeed, leptin inhibition was shown to have several beneficial effects. A leptin peptide functioning as an antagonist mutant leptin reduced food intake when administered intracerebroventricularly [41]. Moreover, a leptin antagonist was shown to reverse hypertension induced by leptin central overexpression [42] and was proposed to be a novel therapeutic approach for the treatment of autoimmune diseases [43].

Given that leptin levels are increased in obesity and play an important proinflammatory role, strategies to attenuate leptin's effects on the immune system would be of great importance. Because leptin is a central negative regulator of body weight, it has been proposed as a treatment for obesity. However, obese individuals exhibit increased circulating leptin levels, and leptin does not reduce food intake or improve any metabolic parameter in these individuals due to the occurrence of leptin resistance in the brain [44]. Moreover, exogenous administration of leptin did not affect appetite or body weight in obese patients, again due to central leptin resistance [45]. However, it was recently described that amylin, another adipokine, can restore leptin sensitivity when combined with leptin, enhancing body weight reduction in obese rodents and humans [46]. It is important to note that leptin administration may exert deleterious effects on the immune system due to its proinflammatory function. Reducing leptin levels could reduce adipose tissue inflammation and the ongoing inflammation that occurs in autoimmune diseases, but it also affects appetite and food intake. Leptin antagonist also may have some therapeutic values and efficient leptin antagonism can be achieved in vivo [47]. A novel antagonist of leptin, produced by pegylation in order to increase the leptin antagonist half-life, increased food intake and weight gain in mice [48]. This weight changes were shown to be reversible once the leptin antagonist treatment was ceased, indicating that this compound could be useful therapeutically for the treatment of cachexia and other metabolic and immune-mediated diseases. Indeed, a monoclonal antibody against leptin receptor was shown to act as an antagonist, blocking human monocyte TNF secretion and anti-CD3 induced T cell proliferation in peripheral blood mononuclear cultures [49]. In summary, the investigation of leptin has shown that even if the mechanism of action is well established with a proven treatment concept in animal models, an efficacious and successful treatment in humans is not guaranteed, albeit leptin antagonism may appear as a new therapeutic target once more human data is obtained.

## 3. Adiponectin

Adiponectin is an adipose tissue-derived cytokine with homology to type VIII and type X collagens and to complement factor C1q. This adipokine is found in the peripheral blood in high concentrations and is present as several molecular isoforms [50]. Adiponectin signals through two receptors, AdipoR1 (expressed preferentially in skeletal muscle) and AdipoR2 (expressed in the liver). The binding of adiponectin to AdipoR1 and R2 leads to the activation of AMPK and PPAR*γ*, as well as other molecules [51]. In the liver, adiponectin increases fatty acid oxidation and reduces liver glucose synthesis. Lean adiponectin knockout mice do not exhibit any severe alteration; but under a high fat diet, these mice display severe insulin resistance and lipid accumulation in muscle. Serum adiponectin is lower in morbidly obese individuals, while its serum levels increase during weight loss or with drugs that enhance insulin sensitivity, such as thiazolidinediones [50, 52].

Adiponectin plays a prominent role in cardiovascular diseases, type 2 diabetes, and metabolic syndromes [53, 54] (Figure 2). Adiponectin exerts relevant actions on both the innate and adaptive immune systems. There is a consensus that adiponectin exerts insulin-sensitizing, anti-inflammatory, and antiapoptotic effects on several cell types [55]. It inhibits phagocytic activity and the production of IL-6 and TNF by macrophages. Adiponectin can induce the production of anti-inflammatory mediators such as IL-10 and IL-1RA by human monocytes, DCs, and macrophages [14]. In T cells, adiponectin receptors are coexpressed with negative T cell regulators, such as CTLA-4 (cytotoxic T-lymphocyte antigen 4) and TIRC7 (T cell immune response c-DNA). Similar to CTLA-4 binding, which results in the blocked of costimulatory stimulus in T cells required for the induction of proliferation, the addition of adiponectin results in decreased antigen-specific T cell expansion and cytokine production [56]. Piccio and coworkers showed that a lack of adiponectin is associated with severe EAE development. This worse EAE is accompanied with higher production of IFN-*γ*, IL-17, TNF-*α*, and IL-6 by the T cells from Adipo^−/−^ mice immunized with myelin antigen [57]. These data suggest that adiponectin may affect T cell responses by lowering the costimulatory stimulus delivered to T cells by antigen-presenting cells. Also, due to the abundance of adiponectin in human plasma, adiponectin appears to play a central role in the systemic regulation of T cell responses.

Adiponectin secretion from fat cells is reduced under adverse metabolic conditions, leading to decreased adiponectin serum levels. Also, several hormones associated with insulin resistance and obesity, such as insulin, catecholamines, TNF, and other proinflammatory cytokines, downregulate the expression of adiponectin in adipocytes, resulting in decreased adiponectin serum levels [58]. The role of adiponectin as an endogenous insulin sensitizer was described in adiponectin knockout mice, which have impaired insulin sensitivity [59]. Moreover, the overexpression of adiponectin in mice is sufficient to improve insulin sensitivity in high fat diet-induced obesity mice models [60, 61]. Recombinant adiponectin administration is sufficient to improve glucose, lipid, and insulin plasma levels as well as insulin receptor expression and liver steatosis in high fat diet-induced obesity mice treated with streptozotocin [62]. This indicates that adiponectin may ameliorate type 1 diabetes and other obesity-related diseases. Recently, an orally active adiponectin receptor agonist was shown to improve insulin resistance and glucose intolerance in mice [63]. This molecule, called AdipoRon, binds to the adiponectin receptor and ameliorates diabetes and prolongs the lifespan of obese mice. Thus, although the anti-inflammatory properties of adiponectin treatment still require further investigation, adiponectin or adiponectin receptor agonists are promising targets for the development of therapeutic drugs to treat insulin-resistant states, particularly because adiponectin also possesses anti-inflammatory properties, which can play an important role in the treatment of adipose tissue inflammation in obese individuals.

## 4. Retinol Binding Protein 4 (RBP4)

RBP4 is a 21-kDa protein that transports retinol from its main storage site in the liver to various tissues in the body. RBP4 is secreted from adipose tissue and the liver. STRA6 (stimulated by retinoic acid 6), a transmembrane protein, is the only characterized RBP4 receptor [64]. STRA6 mediates retinol uptake from retinol-RBP4 and the bidirectional transport of retinol [65]. RBP4 levels are elevated in many insulin-resistant states in mice and humans [66, 67]. RBP4 is the only specific transport protein for retinol (vitamin A) in the circulation, and elevation of serum RBP4 causes systemic insulin resistance [68]. Many studies have shown that serum RBP4 levels correlate with several components of metabolic syndromes in humans, including hypertension [67, 69], dyslipidemia [69, 70], cardiovascular disease [71], and intra-abdominal fat mass [71–73]. RBP4 is elevated in association with insulin resistance in diet-induced obesity and genetically obese (ob/ob) mouse models [68, 74]. Treatment with the insulin sensitizer rosiglitazone lowers serum and adipose RBP4 levels and improves insulin sensitivity [68]. Moreover, whole-body deletion of RBP4 improves insulin sensitivity in mouse models, and treatment with the synthetic retinoid fenretinide lowers serum RBP4 levels by disrupting TTR (transthyretin) interaction with RBP4 and enhancing RBP4 clearance. Lowering RBP4 levels through TTR inhibition was recently shown to be effective, improving insulin resistance and adipose tissue inflammation in obese mice [75]. Additionally, fenretinide is able to improve insulin sensitivity in mice on a high fat diet [68, 76]. Conversely, transgenic overexpression of RBP4 or injections of purified RBP4 into normal mice cause insulin resistance [68, 77]. Mechanistically, RBP4 impairs insulin signaling in muscle by decreasing insulin-stimulated tyrosine phosphorylation of insulin receptor substrate (IRS-1) and PI(3) kinase activity [68]. Elevated serum RBP4 induces insulin resistance in the liver by increasing the expression of phosphoenolpyruvate decarboxylase (PEPCK) and hepatic glucose output [68]. In primary human adipocytes, RBP4 impairs insulin-stimulated IRS1 and ERK1/2 phosphorylation [78].

As mentioned, elevated serum RBP4 levels correlate roughly with insulin resistance [79, 80] and several parameters of metabolic syndromes in humans, including body mass index, intra-abdominal fat mass, systolic blood pressure, serum triglycerides, and decreased high-density lipoproteins, even in large epidemiological studies [67, 81]. Treatment of obesity and/or diabetes through weight loss [82, 83], exercise, lifestyle intervention, bariatric surgery [82], or thiazolidinedione treatment [84] lowers serum RBP4 levels. Moreover, genetic studies have identified an RBP4 promoter polymorphism that increases RBP4 expression [79] and is associated with a ~2-fold increased risk for type 2 diabetes, suggesting that elevated RBP4 levels contribute to diabetes in certain human populations [80].

A recent study has linked RBP4 to inflammation [85]. RBP4 treatment of bone marrow-derived macrophages stimulated TNF and IL-6 release. Depletion of Toll-like receptor 4 (TLR4) from bone marrow-derived macrophages blocked TNF and IL-6 production after RBP4 stimulation [85]. TLR4 is expressed in macrophages and upon stimulation activates NF*κ*B and JNK signaling, causing the release of inflammatory cytokines [86]. Emerging evidence suggests possible roles for proinflammatory pathways in RBP4-induced insulin resistance [77, 87]. RBP4 overexpression induces AT inflammation through the activation of both the innate and adaptive arms of the immune response [77]. RBP4 directly activates antigen-presenting cells, leading to adipose tissue inflammation. This RBP4-induced APC activation results in proinflammatory CD4^+^ T cell proliferation and Th1 polarization (Th1), which further orchestrates adipose tissue inflammation (mononuclear cell infiltration and proinflammatory cytokine production) and, therefore, insulin resistance [77]. These data suggest that approaches to lower RBP4 levels, such as utilizing fenretinide or thiazolidinedione, could lead to new strategies to attenuate adipose tissue inflammation, ameliorating metabolic parameters and insulin resistance. No human data exist regarding approaches to reduce RBP4 levels, and therefore, further investigation is required.

## 5. Visfatin

Visfatin was identified in the liver, skeletal muscle, and bone marrow as a growth factor for B cell precursors and a pre-B-colony enhancing factor (PBEF). Circulating visfatin level reflects white adipose tissue mass and increases with the differentiation of adipocytes. Visfatin transcription is regulated by TNF, IL-6, and glucocorticoids. Importantly, this adipokine is not exclusively produced by adipose tissue [88]. Neutrophils can produce visfatin after being stimulated with endotoxin [88]. Individuals with inflammatory diseases have elevated levels of circulating visfatin [89]. In monocytes, visfatin stimulates TNF, IL-6, and IL-1*β* secretion and costimulatory molecules expression. Visfatin also contributes to macrophage differentiation and cytokine release in a JNK- and NF*κ*B-dependent manner [90]. Additionally, visfatin augments monocyte-induced alloresponses in lymphocytes [91]. Therefore, visfatin is a proinflammatory mediator and might participate in a variety of inflammatory conditions, such as autoimmune diseases and adipose tissue inflammation-induced insulin resistance (Figure 2). Several studies have evaluated the immunological role of visfatin, and its effects on the treatment of inflammatory disorders are now known. For example, FK866, a visfatin inhibitor, attenuates intestinal and lung injury by inhibiting proinflammatory cytokine production and NF*κ*B activation, leading to improved survival rate [92]. Moreover, FK866 damps CXCL2-induced neutrophil recruitment, reducing neutrophil-mediated tissue injury in mice [93]. This could be of great importance for the treatment of acute inflammatory diseases, such as sepsis. Also, visfatin has an important enzymatic role in the synthesis of nicotinamide mononucleotide (NMN). Aging and hypercaloric feeding compromise NAMPT- (nicotinamide phosphoribosyl transferase-) mediated NAD^+^ biosynthesis and may contribute to the pathogenesis of type 2 diabetes [94]. Albeit the role of visfatin in inflammation seems promising, few human studies have been conducted exploring visfatin as a therapeutic target.

## 6. TNF

TNF is a master cytokine that mediates many inflammatory responses and is implicated in the pathogenesis of several diseases, including cancer, sepsis, rheumatoid arthritis, diabetes, and inflammatory bowel disease [95–97]. Because TNF is secreted by the adipose tissue in obese mice and humans, TNF is also considered an adipokine [98, 99]. The major pathways activated by TNF include caspases, NF*κ*B, and mitogen-activated protein kinases (MAP kinases). Functional interaction between these signaling pathways can define the physiological outcome of a TNF-induced response. It is important to know that TNF acts in two waves, which contributes to the biological activity of TNF [100]. During the first phase, TNF signaling induces expression of inflammatory cytokines. These inflammatory cytokines initiate a secondary cellular response. The biphasic nature of TNF signaling complicates the analysis of the TNF signaling pathway and its application as a drug target. For example, the MAP kinases activated by TNF increase the expression of TNF by target cells while the MAP kinases act both upstream and downstream of TNF signaling.

In obese rats, the neutralization of TNF resulted in increased peripheral glucose uptake induced by insulin [101], and mice with a TNF gene deletion have significant improvement in their insulin sensitivity in both monogenetic and diet-induced obesity models [99]. These data support the notion that blocking TNF may result in improved insulin sensitivity and its related benefits. Indeed the administration of anti-TNF antibodies in mice resulted in reduced inflammation and the consequent protection against diet-induced obesity and insulin resistance [102, 103]. However, in obese Zucker rats, anti-TNF treatment did not affect insulin resistance [104]. Moreover, the successful improvement of metabolic diseases with anti-TNF treatment observed in some animal models failed to be effective in clinical trials. Treatment of human subjects with anti-TNF antibodies, such as Infliximab or Etanercept, did not improve insulin resistance or improved obesity [105, 106]. However, a few studies reported improvement in insulin sensitivity and glucose homeostasis during prolonged treatment with Infliximab [107] or Etanercept [108]. Importantly, albeit chronic treatment with Infliximab improved inflammatory status, it did not affect insulin sensitivity in diabetic obese individuals [106]. Among the possible causes of the lack of effect seen with anti-TNF treatment in insulin-resistant obese individuals, one may be the paracrine action of TNF [109]. Also, because Infliximab is primarily distributed in the vascular compartment, its effectiveness in peripheral tissues, such as adipose tissue, may be low. Despite some promising preclinical trials, the lack of human data is a major problem with the use of TNF antagonists as new therapeutic approaches. Thus far, TNF appears not to be a promising drug target, at least for the treatment of insulin resistance in humans.

## 7. IL-1***β***

IL-1*β* is expressed and secreted by adipose tissue and is a proinflammatory cytokine that plays an important role in pancreatic *β*-cell destruction in type 1 diabetes [110, 111]. Due to the enormous attention raised by the description of the NLRP3 (NOD-like receptor family, pyrin domain containing 3) inflammasome [112, 113] and by the dramatic response to IL-1*β* blockers observed in patients with a gain of function mutation for the NLRP3 gene (cryopyrin-associated periodic syndromes (CAPSs)) [114, 115], IL-1*β* is now considered one of most important proinflammatory cytokines in several inflammatory diseases. The deployment of specific IL-1-targeting agents has demonstrated a pathological role for IL-1*β*-mediated inflammation in a growing list of inflammatory diseases.

IL-1*β* is produced by a limited number of immune cells, including monocytes, macrophages, and dendritic cells. Importantly, IL-1*β* is inactive under steady state conditions and requires several intracellular events to be cleaved into an active and secreted form. The search for targeting IL-1 began early in the 90s with the identification of a naturally occurring IL-1 receptor antagonist (IL-1Ra, Anakinra), which blocks both IL-1*α* and IL-1*β* activities. IL-1Ra competes with free IL-1*α* and IL-1*β* for IL-1R1 binding, preventing signal transduction. Due to its short half-life it is administered daily. The blockade of IL-1 with Anakinra improved glycaemia, *β*-cell function, and circulating inflammatory factors in a clinical trial involving 70 patients with type 2 diabetes [110, 116]. This showed that IL-1 blockers could have a high impact in the treatment of obesity-induced inflammation and insulin resistance as well as the treatment of autoimmune diseases, such as type 1 diabetes. In addition to Anakinra, some other IL-1R blockers were developed. Rilonacept, a protein with the extracellular domains of humanized IL-1 receptor and IL-1 receptor accessory protein fused to the Fc portion of an IgG1, displays a better half-life compared to Anakinra, being administered once a week. Rilonacept binds to IL-1*β* and IL-1*α* with high affinity, inhibiting IL-1 activity [117], and was shown to be effective in the treatment of gout [117, 118]. Another IL-1R blocker, Canakinumab, is a human anti-IL-1*β* monoclonal antibody. Importantly, different from previous blockers, Canakinumab does not cross-react with IL-1*α* or IL-1Ra. Thus, Canakinumab specifically prevents the interaction of IL-1*β* with its receptor, which results in blockage of the inflammatory signaling cascade and has an even longer half-life compared to other compounds. In humans, Canakinumab proved to be effective in the treatment of several inflammatory diseases [119, 120]. Another recently described compound, Gevokizumab, is an IgG2 humanized monoclonal antibody. Gevokizumab has a different action compared to the IL-1 blockers described above, Gevokizumab modulates IL-1*β* bioactivity by reducing the affinity of IL-1*β* for the IL-1RI:IL-1RAcP signaling complex [121]. This molecule has a very long half-life and can be administered once a month. Clinical trials are ongoing for osteoarthritis, noninfectious uveitis, and diabetes mellitus [121, 122]. Taken together, IL-1*β* represents a model in which adipokines may be indirectly employed as target molecules for the treatment of obesity-related and inflammation-induced insulin resistance comorbidities as well as for some autoimmune diseases.

## 8. Other Adipokines

In addition to the adipokines mentioned above, several others have been identified; their roles in immune-mediated diseases are not entirely understood and are currently being explored. Some of these factors include apelin, vaspin, and hepcidin [123]. Apelin was identified as a peptide and an endogenous binder of the orphan G-protein-coupled receptor APJ (apelin receptor). Apelin secretion in adipose tissue is increased by inflammatory factors, such as by TNF. In diet-induced obese mice, the levels of proinflammatory factors and the macrophage cell number increase as the adipose tissue expands, and it is possible that apelin plays a role in promoting this condition. Although there is a lack of information regarding the participation of apelin in immune responses, some data support its participation in tumor neovascularization, since apelin promotes the proliferation of endothelial cells [124]. The treatment of rats with apelin receptor antagonist lowered hepatic fibrosis, which suggests a beneficial role for apelin targeting in the liver [125]. More recently, the inhibition of apelin with pharmacology blockader (F13A) increased liver regeneration after hepatectomy, strengthening the potential importance of apelin in liver diseases [126]. In obese and insulin-resistant mice, the injection of recombinant apelin results in enhanced glucose utilization in skeletal muscle and the restoration of glucose tolerance [127]. Data regarding apelin obtained from different animal models indicate that apelin influences glucose homeostasis and may contribute to the link between increased adipose tissue mass and obesity-related metabolic and maybe inflammatory diseases. However, the precise mechanisms by which apelin exerts beneficial effects on glucose metabolism still require further investigation in humans.

Vaspin (visceral adipose tissue-derived serine protease inhibitor) was first described in 2005 as a serine protease inhibitor produced by visceral adipose tissue [128]. The administration of vaspin to obese mice improved glucose tolerance and insulin sensitivity [128]. The induction of vaspin by adipose tissue appears to constitute a compensatory mechanism in response to obesity. Administration of vaspin to obese mice improves glucose tolerance and insulin sensitivity and modifies the expression of genes involved in insulin resistance [129]. Moreover, treatment of different animal models with vaspin led to sustained decreased glucose levels and lower food intake [130]. Interestingly, vaspin is modulated by the energy status of the placenta, indicating that this protein may be involved in the regulation of placental metabolic functions [131]. These data suggest that vaspin is a promising pharmacological agent for the treatment of obesity and its related metabolic complications.

Hepcidin, described in 2001 as an antimicrobial urinary peptide produced by liver, was later characterized as an adipokine [132]. Hepcidin is an important regulator of iron homeostasis. The production of hepcidin does not exclusively depend on iron metabolism, but it can be stimulated by hypoxia and inflammatory stimuli [133]. The levels of this adipokine are higher in disorders involving generalized inflammation that results in hypoferremia. In mice with acute inflammation, hepcidin production is stimulated by IL-6 and by the STAT3 pathway [134]. Additionally, hepcidin acts against invading microorganisms by decreasing extracellular iron levels, limiting the amount of iron available to the microorganisms. Since hepcidin is induced by IL-6 and STAT3, it may also be induced by leptin, and if so, a higher body mass index and obesity could lead to elevated production of hepcidin. In some pathological conditions, hepcidin levels are inadequately elevated and reduce iron availability in the body, which leads to anemia. These elevated hepcidin levels are observed in conditions such as common anemia of chronic disease (ACD) or anemia of inflammation. To date, no definitive treatment for ACD exists. The agents utilized to treat this condition are barely effective and may have adverse side effects. Alternative approaches aimed at pharmacologically controlling the expression of hepcidin by targeting different regulatory steps have been attempted. These include hepcidin-sequestering agents, inhibitors of the IL6/STAT3 pathway or hepcidin transduction (siRNA/shRNA), and ferroportin stabilizers [135]. Although most agents have only been evaluated in preclinical studies, several have reached human clinical trials. Although the clinical effectiveness of hepcidin-targeted therapies has yet to be established, there is optimism that hepcidin agonists and antagonists will improve the treatment of patients with iron disorders, either alone or in combination with existing therapies.

## 9. Conclusions

Antiobesity treatments have offered only limited long-term success (lifestyle changes, physical activities, diets, and pharmacotherapies) or are associated with a relatively high mortality risk (bariatric surgery). Thus, there is an increased need for the development of new strategies to pharmacologically treat the health problems associated with obesity and chronic inflammation. Because adipokines are involved in the regulation of appetite, satiety, energy expenditure, inflammation, and physical activities, they may represent important targets for therapeutic interventions in the treatment of chronic inflammatory diseases, such as obesity and autoimmune diseases. A lack of understanding of the immunological roles and mechanisms of adipokine actions and their potential side effects are still problems to be solved in drug discovery. New adipokine-targeting compounds and treatment strategies may offer exciting new approaches for a spectrum of diseases with multiple unmet clinical needs.

## References

[1] Guh D. P., Zhang W., Bansback N., Amarsi Z., Birmingham C. L., Anis A. H. (2009). The incidence of co-morbidities related to obesity and overweight: a systematic review and meta-analysis. *BMC Public Health*.

[2] Sauvanet J. P. (2003). Congress of the International Diabetes Federation (IDF-Paris 2003). *La Presse Médicale*.

[3] Ratner R. E. (1998). Type 2 diabetes mellitus: the grand overview. *Diabetic Medicine*.

[4] Moraes-Vieira P. M. M., Bassi Ê. J., Araujo R. C., Câmara N. O. S. (2012). Leptin as a link between the immune system and kidney-related diseases: leading actor or just a coadjuvant?. *Obesity Reviews*.

[5] Kershaw E. E., Flier J. S. (2004). Adipose tissue as an endocrine organ. *The Journal of Clinical Endocrinology & Metabolism*.

[6] Ahima R. S. (2006). Adipose tissue as an endocrine organ. *Obesity*.

[7] La Cava A., Matarese G. (2004). The weight of leptin in immunity. *Nature Reviews Immunology*.

[8] Zhang Y., Proenca R., Maffei M., Barone M., Leopold L., Friedman J. M. (1994). Positional cloning of the mouse *obese* gene and its human homologue. *Nature*.

[9] Flier J. S. (1995). The adipocyte: storage depot or node on the energy information superhighway?. *Cell*.

[10] Friedman J. M., Halaas J. L. (1998). Leptin and the regulation of body weight in mammals. *Nature*.

[11] Villanueva E. C., Myers M. G. (2008). Leptin receptor signaling and the regulation of mammalian physiology. *International Journal of Obesity*.

[12] Howard J. K., Lord G. M., Matarese G. (1999). Leptin protects mice from starvation-induced lymphoid atrophy and increases thymic cellularity in ob/ob mice. *Journal of Clinical Investigation*.

[13] Moraes-Vieira P. M., Larocca R. A., Bassi E. J. (2014). Leptin deficiency impairs maturation of dendritic cells and enhances induction of regulatory T and Th17 cells. *European Journal of Immunology*.

[14] Tilg H., Moschen A. R. (2006). Adipocytokines: mediators linking adipose tissue, inflammation and immunity. *Nature Reviews Immunology*.

[15] Jitprasertwong P., Jaedicke K. M., Nile C. J., Preshaw P. M., Taylor J. J. (2014). Leptin enhances the secretion of interleukin (IL)-18, but not IL-1*β*, from human monocytes via activation of caspase-1. *Cytokine*.

[16] Mancuso P., Gottschalk A., Phare S. M., Peters-Golden M., Lukacs N. W., Huffnagle G. B. (2002). Leptin-deficient mice exhibit impaired host defense in Gram-negative pneumonia. *Journal of Immunology*.

[17] Zarkesh-Esfahani H., Pockley G., Metcalfe R. A. (2001). High-dose leptin activates human leukocytes via receptor expression on monocytes. *The Journal of Immunology*.

[18] Santos-Alvarez J., Goberna R., Sánchez-Margalet V. (1999). Human leptin stimulates proliferation and activation of human circulating monocytes. *Cellular Immunology*.

[19] Amarilyo G., Iikuni N., Liu A., Matarese G., La Cava A., Bobé P. (2014). Leptin enhances availability of apoptotic cell-derived self-antigen in systemic lupus erythematosus. *PLoS ONE*.

[20] Acedo S. C., Gambero S., Cunha F. G. P., Lorand-Metze I., Gambero A. (2013). Participation of leptin in the determination of the macrophage phenotype: an additional role in adipocyte and macrophage crosstalk. *In Vitro Cellular & Developmental Biology—Animal*.

[21] Caldefie-Chezet F., Poulin A., Tridon A., Sion B., Vasson M.-P. (2001). Leptin: a potential regulator of polymorphonuclear neutrophil bactericidal action?. *Journal of Leukocyte Biology*.

[22] Caldefie-Chezet F., Poulin A., Vasson M.-P. (2003). Leptin regulates functional capacities of polymorphonuclear neutrophils. *Free Radical Research*.

[23] Tian Z., Sun R., Wei H., Gao B. (2002). Impaired natural killer (NK) cell activity in leptin receptor deficient mice: leptin as a critical regulator in NK cell development and activation. *Biochemical and Biophysical Research Communications*.

[24] Zhao Y., Sun R., You L., Gao C., Tian Z. (2003). Expression of leptin receptors and response to leptin stimulation of human natural killer cell lines. *Biochemical and Biophysical Research Communications*.

[25] Toniolo P. A., Liu S., Yeh J. E. (2015). Inhibiting STAT5 by the BET bromodomain inhibitor JQ1 disrupts human dendritic cell maturation. *The Journal of Immunology*.

[26] Mattioli B., Straface E., Quaranta M. G., Giordani L., Viora M. (2005). Leptin promotes differentiation and survival of human dendritic cells and licenses them for Th1 priming. *The Journal of Immunology*.

[27] Lam Q. L. K., Liu S., Cao X., Lu L. (2006). Involvement of leptin signaling in the survival and maturation of bone marrow-derived dendritic cells. *European Journal of Immunology*.

[28] Ramirez O., Garza K. M. (2014). Leptin deficiency in vivo enhances the ability of splenic dendritic cells to activate T cells. *International Immunology*.

[29] Lord G. M., Matarese G., Howard J. K., Baker R. J., Bloom S. R., Lechler R. I. (1998). Leptin modulates the T-cell immune response and reverses starvation-induced immunosuppression. *Nature*.

[30] Martín-Romero C., Santos-Alvarez J., Goberna R., Sánchez-Margalet V. (2000). Human leptin enhances activation and proliferation of human circulating T lymphocytes. *Cellular Immunology*.

[31] Batra A., Okur B., Glauben R. (2010). Leptin: a critical regulator of CD4^+^ T-cell polarization in vitro and in vivo. *Endocrinology*.

[32] de Rosa V., Procaccini C., Calì G. (2007). A key role of leptin in the control of regulatory T cell proliferation. *Immunity*.

[33] Taleb S., Herbin O., Ait-Oufella H. (2007). Defective leptin/leptin receptor signaling improves regulatory T cell immune response and protects mice from atherosclerosis. *Arteriosclerosis, Thrombosis, and Vascular Biology*.

[34] Moraes-Vieira P. M. M., Bassi E. J., Larocca R. A. (2013). Leptin modulates allograft survival by favoring a Th2 and a regulatory immune profile. *The American Journal of Transplantation*.

[35] Faggioni R., Jones-Carson J., Reed D. A. (2000). Leptin-deficient (*ob*/*ob*) mice are protected from T cell-mediated hepatotoxicity: role of tumor necrosis factor *α* and IL-18. *Proceedings of the National Academy of Sciences of the United States of America*.

[36] Matarese G., Procaccini C., De Rosa V. (2008). The intricate interface between immune and metabolic regulation: a role for leptin in the pathogenesis of multiple sclerosis?. *Journal of Leukocyte Biology*.

[37] Saucillo D. C., Gerriets V. A., Sheng J., Rathmell J. C., MacIver N. J. (2014). Leptin metabolically licenses T cells for activation to link nutrition and immunity. *The Journal of Immunology*.

[38] Macintyre A. N., Gerriets V. A., Nichols A. G. (2014). The glucose transporter Glut1 is selectively essential for CD4 T cell activation and effector function. *Cell Metabolism*.

[39] Deng J., Liu Y., Yang M. (2012). Leptin exacerbates collagen-induced arthritis via enhancement of Th17 cell response. *Arthritis and Rheumatism*.

[40] Fujita Y., Fujii T., Mimori T. (2014). Deficient leptin signaling ameliorates systemic lupus erythematosus lesions in MRL/Mp-Fasl pr mice. *Journal of Immunology*.

[41] Brunner L., Whitebread S., Leconte I. (1999). A peptide leptin antagonist reduces food intake in rodents. *International Journal of Obesity*.

[42] Tümer N., Erdös B., Matheny M., Cudykier I., Scarpace P. J. (2007). Leptin antagonist reverses hypertension caused by leptin overexpression, but fails to normalize obesity-related hypertension. *Journal of Hypertension*.

[43] Babaei A., Zarkesh-Esfahani S. H., Bahrami E., Ross R. J. (2011). Restricted leptin antagonism as a therapeutic approach to treatment of autoimmune diseases. *Hormones*.

[44] Ahima R. S., Flier J. S. (2000). Adipose tissue as an endocrine organ. *Trends in Endocrinology and Metabolism*.

[45] Savage D. B., O'Rahilly S. (2002). Leptin: a novel therapeutic role in lipodystrophy. *Journal of Clinical Investigation*.

[46] Roth J. D., Roland B. L., Cole R. L. (2008). Leptin responsiveness restored by amylin agonism in diet-induced obesity: evidence from nonclinical and clinical studies. *Proceedings of the National Academy of Sciences of the United States of America*.

[47] Shpilman M., Niv-Spector L., Katz M. (2011). Development and characterization of high affinity leptins and leptin antagonists. *The Journal of Biological Chemistry*.

[48] Elinav E., Niv-Spector L., Katz M. (2009). Pegylated leptin antagonist is a potent orexigenic agent: preparation and mechanism of activity. *Endocrinology*.

[49] Fazeli M., Zarkesh-Esfahani H., Wu Z. (2006). Identification of a monoclonal antibody against the leptin receptor that acts as an antagonist and blocks human monocyte and T cell activation. *Journal of Immunological Methods*.

[50] Oh D. K., Ciaraldi T., Henry R. R. (2007). Adiponectin in health and disease. *Diabetes, Obesity and Metabolism*.

[51] Kadowaki T., Yamauchi T., Kubota N. (2008). The physiological and pathophysiological role of adiponectin and adiponectin receptors in the peripheral tissues and CNS. *FEBS Letters*.

[52] Kadowaki T., Yamauchi T. (2005). Adiponectin and adiponectin receptors. *Endocrine Reviews*.

[53] Bik W., Baranowska B. (2009). Adiponectin—a predictor of higher mortality in cardiovascular disease or a factor contributing to longer life?. *Neuroendocrinology Letters*.

[54] Swellam M., Mahmoud M. S., Ali A. A.-F. (2009). Clinical implications of adiponectin and inflammatory biomarkers in type 2 diabetes mellitus. *Disease Markers*.

[55] Turer A. T., Scherer P. E. (2012). Adiponectin: mechanistic insights and clinical implications. *Diabetologia*.

[56] Wilk S., Scheibenbogen C., Bauer S. (2011). Adiponectin is a negative regulator of antigen-activated T cells. *European Journal of Immunology*.

[57] Piccio L., Cantoni C., Henderson J. G. (2013). Lack of adiponectin leads to increased lymphocyte activation and increased disease severity in a mouse model of multiple sclerosis. *European Journal of Immunology*.

[58] Duntas L. H., Popovic V., Panotopoulos G. (2004). Adiponectin: novelties in metabolism and hormonal regulation. *Nutritional Neuroscience*.

[59] Fruebis J., Tsao T.-S., Javorschi S. (2001). Proteolytic cleavage product of 30-kDa adipocyte complement-related protein increases fatty acid oxidation in muscle and causes weight loss in mice. *Proceedings of the National Academy of Sciences of the United States of America*.

[60] Bauche I. B., El Mkadem S. A., Pottier A.-M. (2007). Overexpression of adiponectin targeted to adipose tissue in transgenic mice: impaired adipocyte differentiation. *Endocrinology*.

[61] Yamauchi T., Kamon J., Waki H. (2003). Globular adiponectin protected ob/ob mice from diabetes and ApoE-deficient mice from atherosclerosis. *The Journal of Biological Chemistry*.

[62] Ma H., Cui F., Dong J. J. (2014). Therapeutic effects of globular adiponectin in diabetic rats with nonalcoholic fatty liver disease. *World Journal of Gastroenterology*.

[63] Okada-Iwabu M., Yamauchi T., Iwabu M. (2013). A small-molecule AdipoR agonist for type 2 diabetes and short life in obesity. *Nature*.

[64] Kawaguchi R., Yu J., Honda J. (2007). A membrane receptor for retinol binding protein mediates cellular uptake of vitamin A. *Science*.

[65] Isken A., Golczak M., Oberhauser V. (2008). RBP4 disrupts vitamin A uptake homeostasis in a STRA6-deficient animal model for Matthew-Wood syndrome. *Cell Metabolism*.

[66] Sun Q., Kiernan U. A., Shi L. (2013). Plasma retinol-binding protein 4 (RBP4) levels and risk of coronary heart disease: a prospective analysis among women in the nurses' health study. *Circulation*.

[67] Qi Q., Yu Z., Ye X. (2007). Elevated retinol-binding protein 4 levels are associated with metabolic syndrome in Chinese people. *Journal of Clinical Endocrinology & Metabolism*.

[68] Yang Q., Graham T. E., Mody N. (2005). Serum retinol binding protein 4 contributes to insulin resistance in obesity and type 2 diabetes. *Nature*.

[69] Yang Q., Eskurza I., Kiernan U. A. (2012). Quantitative measurement of full-length and C-terminal proteolyzed RBP4 in serum of normal and insulin-resistant humans using a novel mass spectrometry immunoassay. *Endocrinology*.

[70] Yoshida A., Matsutani Y., Fukuchi Y., Saito K., Naito M. (2006). Analysis of the factors contributing to serum retinol binding protein and iransthyretin leveis in Japanese adults. *Journal of Atherosclerosis and Thrombosis*.

[71] Ingelsson E., Sundström J., Melhus H. (2009). Circulating retinol-binding protein 4, cardiovascular risk factors and prevalent cardiovascular disease in elderly. *Atherosclerosis*.

[72] Tschoner A., Sturm W., Engl J. (2008). Retinol-binding protein 4, visceral fat, and the metabolic syndrome: effects of weight loss. *Obesity*.

[73] Park S. E., Lee N. S., Park J. W. (2014). Association of urinary RBP4 with insulin resistance, inflammation, and microalbuminuria. *European Journal of Endocrinology*.

[74] Mody N., Graham T. E., Tsuji Y., Yang Q., Kahn B. B. (2008). Decreased clearance of serum retinol-binding protein and elevated levels of transthyretin in insulin-resistant ob/ob mice. *The American Journal of Physiology—Endocrinology and Metabolism*.

[75] Zemany L., Bhanot S., Peroni O. D. (2014). Transthyretin antisense oligonucleotides lower circulating RBP4 levels and improve insulin sensitivity in obese mice. *Diabetes*.

[76] Preitner F., Mody N., Graham T. E., Peroni O. D., Kahn B. B. (2009). Long-term Fenretinide treatment prevents high-fat diet-induced obesity, insulin resistance, and hepatic steatosis. *American Journal of Physiology—Endocrinology and Metabolism*.

[77] Moraes-Vieira P., Yore M., Dwyer P., Syed I., Aryal P., Kahn B. (2014). RBP4 activates antigen-presenting cells, leading to adipose tissue inflammation and systemic insulin resistance. *Cell Metabolism*.

[78] Öst A., Danielsson A., Lidén M., Eriksson U., Nystrom F. H., Strålfors P. (2007). Retinol-binding protein-4 attenuates insulin-induced phosphorylation of IRS1 and ERK1/2 in primary human adipocytes. *The FASEB Journal*.

[79] Munkhtulga L., Nakayama K., Utsumi N. (2007). Identification of a regulatory SNP in the retinol binding protein 4 gene associated with type 2 diabetes in Mongolia. *Human Genetics*.

[80] van Hoek M., Dehghan A., Zillikens M. C., Hofman A., Witteman J. C., Sijbrands E. J. G. (2008). An RBP4 promoter polymorphism increases risk of type 2 diabetes. *Diabetologia*.

[81] Graham T. E., Yang Q., Blüher M. (2006). Retinol-binding protein 4 and insulin resistance in lean, obese, and diabetic subjects. *The New England Journal of Medicine*.

[82] Haider D. G., Schindler K., Prager G. (2007). Serum retinol-binding protein 4 is reduced after weight loss in morbidly obese subjects. *The Journal of Clinical Endocrinology & Metabolism*.

[83] Reinehr T., Stoffel-Wagner B., Roth C. L. (2008). Retinol-binding protein 4 and its relation to insulin resistance in obese children before and after weight loss. *Journal of Clinical Endocrinology & Metabolism*.

[84] Haider D. G., Schindler K., Mittermayer F. (2007). Effect of rosiglitazone on visfatin and retinol-binding protein-4 plasma concentrations in HIV-positive patients. *Clinical Pharmacology & Therapeutics*.

[85] Deng Z.-B., Poliakov A., Hardy R. W. (2009). Adipose tissue exosome-like vesicles mediate activation of macrophage-induced insulin resistance. *Diabetes*.

[86] Guha M., Mackman N. (2001). LPS induction of gene expression in human monocytes. *Cellular Signalling*.

[87] Norseen J., Hosooka T., Hammarstedt A. (2012). Retinol-binding protein 4 inhibits insulin signaling in adipocytes by inducing proinflammatory cytokines in macrophages through a c-Jun N-terminal kinase- and toll-like receptor 4-dependent and retinol-independent mechanism. *Molecular and Cellular Biology*.

[88] Jia S. H., Li Y., Parodo J. (2004). Pre-B cell colony-enhancing factor inhibits neutrophil apoptosis in experimental inflammation and clinical sepsis. *The Journal of Clinical Investigation*.

[89] Valentini L., Wirth E. K., Schweizer U. (2009). Circulating adipokines and the protective effects of hyperinsulinemia in inflammatory bowel disease. *Nutrition*.

[90] Yun M. R., Seo J. M., Park H. Y. (2014). Visfatin contributes to the differentiation of monocytes into macrophages through the differential regulation of inflammatory cytokines in THP-1 cells. *Cellular Signalling*.

[91] Moschen A. R., Kaser A., Enrich B. (2007). Visfatin, an adipocytokine with proinflammatory and immunomodulating properties. *Journal of Immunology*.

[92] Matsuda A., Yang W.-L., Jacob A. (2014). FK866, a visfatin inhibitor, protects against acute lung injury after intestinal ischemia-reperfusion in mice via NF-*κ*B pathway. *Annals of Surgery*.

[93] Montecucco F., Bauer I., Braunersreuther V. (2013). Inhibition of nicotinamide phosphoribosyltransferase reduces neutrophil-mediated injury in myocardial infarction. *Antioxidants & Redox Signaling*.

[94] Yoshino J., Mills K. F., Yoon M. J., Imai S.-I. (2011). Nicotinamide mononucleotide, a key NAD^+^ intermediate, treats the pathophysiology of diet- and age-induced diabetes in mice. *Cell Metabolism*.

[95] Wong M., Ziring D., Korin Y. (2008). TNF*α* blockade in human diseases: mechanisms and future directions. *Clinical Immunology*.

[96] Bassi Ê. J., Moraes-Vieira P. M. M., Moreira-Sá C. S. R. (2012). Immune regulatory properties of allogeneic adipose-derived mesenchymal stem cells in the treatment of experimental autoimmune diabetes. *Diabetes*.

[97] Castoldi A., Braga T. T., Correa-Costa M. (2012). TLR2, TLR4 and the Myd88 signaling pathway are crucial for neutrophil migration in acute kidney injury induced by sepsis. *PLoS ONE*.

[98] Hotamisligil G. S., Arner P., Caro J. F., Atkinson R. L., Spiegelman B. M. (1995). Increased adipose tissue expression of tumor necrosis factor-*α* in human obesity and insulin resistance. *Journal of Clinical Investigation*.

[99] Uysal K. T., Wiesbrock S. M., Marino M. W., Hotamisligil G. S. (1997). Protection from obesity-induced insulin resistance in mice lacking TNF-*α* function. *Nature*.

[100] Janes K. A., Gaudet S., Albeck J. G., Nielsen U. B., Lauffenburger D. A., Sorger P. K. (2006). The response of human epithelial cells to TNF involves an inducible autocrine cascade. *Cell*.

[101] Spiegelman B. M., Hotamisligil G. S. (1993). Through thick and thin: wasting, obesity, and TNFalpha. *Cell*.

[102] Li Z., Yang S., Lin H. (2003). Probiotics and antibodies to TNF inhibit inflammatory activity and improve nonalcoholic fatty liver disease. *Hepatology*.

[103] Liang H., Yin B., Zhang H. (2008). Blockade of tumor necrosis factor (TNF) receptor type 1-mediated TNF-*α* signaling protected Wistar rats from diet-induced obesity and insulin resistance. *Endocrinology*.

[104] López-Soriano J., López-Soriano F. J., Bagby G. J., Williamson D. H., Argilés J. M. (1997). Anti-TNF treatment does not reverse the abnormalities in lipid metabolism of the obese Zucker rat. *American Journal of Physiology—Endocrinology and Metabolism*.

[105] Ofei F., Hurel S., Newkirk J., Sopwith M., Taylor R. (1996). Effects of an engineered human anti-TNF-*α* antibody (CDP571) on insulin sensitivity and glycemic control in patients with NIDDM. *Diabetes*.

[106] Wascher T. C., Lindeman J. H. N., Sourij H., Kooistra T., Pacini G., Roden M. (2011). Chronic TNF-*α* neutralization does not improve insulin resistance or endothelial function in ‘healthy’ men with metabolic syndrome. *Molecular Medicine*.

[107] Yazdani-Biuki B., Stelzl H., Brezinschek H. P. (2004). Improvement of insulin sensitivity in insulin resistant subjects during prolonged treatment with the anti-TNF-*α* antibody infliximab. *European Journal of Clinical Investigation*.

[108] Stanley T. L., Zanni M. V., Johnsen S. (2011). TNF-*α* antagonism with etanercept decreases glucose and increases the proportion of high molecular weight adiponectin in obese subjects with features of the metabolic syndrome. *The Journal of Clinical Endocrinology & Metabolism*.

[109] Di Rocco P., Manco M., Rosa G., Greco A. V., Mingrone G. (2004). Lowered tumor necrosis factor receptors, but not increased insulin sensitivity, with infliximab. *Obesity Research*.

[110] Larsen C. M., Faulenbach M., Vaag A. (2007). Interleukin-1-receptor antagonist in type 2 diabetes mellitus. *The New England Journal of Medicine*.

[111] Sopasakis V. R., Nagaev I., Smith U. (2005). Cytokine release from adipose tissue of nonobese individuals. *International Journal of Obesity*.

[112] Gonçalves G. M., Zamboni D. S., Cĝmara N. O. S. (2010). The role of innate immunity in septic acute kidney injuries. *Shock*.

[113] Strowig T., Henao-Mejia J., Elinav E., Flavell R. (2012). Inflammasomes in health and disease. *Nature*.

[114] Hoffman H. M., Rosengren S., Boyle D. L. (2004). Prevention of cold-associated acute inflammation in familial cold autoinflammatory syndrome by interleukin-1 receptor antagonist. *The Lancet*.

[115] Lovell D. J., Bowyer S. L., Solinger A. M. (2005). Interleukin-1 blockade by anakinra improves clinical symptoms in patients with neonatal-onset multisystem inflammatory disease. *Arthritis and Rheumatism*.

[116] Larsen C. M., Faulenbach M., Vaag A., Ehses J. A., Donath M. Y., Mandrup-Poulsen T. (2009). Sustained effects of interleukin-1 receptor antagonist treatment in type 2 diabetes. *Diabetes Care*.

[117] Lovell D. J., Giannini E. H., Reiff A. O. (2013). Long-term safety and efficacy of rilonacept in patients with systemic juvenile idiopathic arthritis. *Arthritis and Rheumatism*.

[118] Mitha E., Ralph Schumacher H., Fouche L. (2013). Rilonacept for gout flare prevention during initiation of uric acid-lowering therapy: results from the PRESURGE-2 international, phase 3, randomized, placebo-controlled trial. *Rheumatology*.

[119] Dinarello C. A., van der Meer J. W. M. (2013). Treating inflammation by blocking interleukin-1 in humans. *Seminars in Immunology*.

[120] Perez-Ruiz F., Chinchilla S. P., Herrero-Beites A. M. (2014). Canakinumab for gout: a specific, patient-profiled indication. *Expert Review of Clinical Immunology*.

[121] Geiler J., McDermott M. F. (2010). Gevokizumab, an anti-IL-1*β* mAb for the potential treatment of type 1 and 2 diabetes, rheumatoid arthritis and cardiovascular disease. *Current Opinion in Molecular Therapeutics*.

[122] Gül A., Tugal-Tutkun I., Dinarello C. A. (2012). Interleukin-1beta-regulating antibody XOMA 052 (gevokizumab) in the treatment of acute exacerbations of resistant uveitis of Behçet's disease: an open-label pilot study. *Annals of the Rheumatic Diseases*.

[123] Balagopal P., Graham T. E., Kahn B. B., Altomare A., Funanage V., George D. (2007). Reduction of elevated serum retinol binding protein in obese children by lifestyle intervention: association with subclinical inflammation. *The Journal of Clinical Endocrinology & Metabolism*.

[124] Masri B., Berghe L. V. D., Sorli C., Knibiehler B., Audigier Y. (2009). Apelin signalisation and vascular physiopathology. *Journal de la Societe de Biologie*.

[125] Principe A., Melgar-Lesmes P., Fernández-Varo G. (2008). The hepatic apelin system: a new therapeutic target for liver disease. *Hepatology*.

[126] Yoshiya S., Shirabe K., Imai D. (2014). Blockade of the apelin-APJ system promotes mouse liver regeneration by activating Kupffer cells after partial hepatectomy. *Journal of Gastroenterology*.

[127] Dray C., Knauf C., Daviaud D. (2008). Apelin stimulates glucose utilization in normal and obese insulin-resistant mice. *Cell Metabolism*.

[128] Hida K., Wada J., Eguchi J. (2005). Visceral adipose tissue-derived serine protease inhibitor: a unique insulin-sensitizing adipocytokine in obesity. *Proceedings of the National Academy of Sciences of the United States of America*.

[129] Heiker J. T., Klöting N., Kovacs P. (2013). Vaspin inhibits kallikrein 7 by serpin mechanism. *Cellular and Molecular Life Sciences*.

[130] Klöting N., Kovacs P., Kern M. (2011). Central vaspin administration acutely reduces food intake and has sustained blood glucose-lowering effects. *Diabetologia*.

[131] Caminos J. E., Bravo S. B., Garcés M. F. (2009). Vaspin and amylin are expressed in human and rat placenta and regulated by nutritional status. *Histology and Histopathology*.

[132] Park C. H., Valore E. V., Waring A. J., Ganz T. (2001). Hepcidin, a urinary antimicrobial peptide synthesized in the liver. *The Journal of Biological Chemistry*.

[133] Fleming R. E. (2007). Hepcidin activation during inflammation: make it STAT. *Gastroenterology*.

[134] Pietrangelo A., Dierssen U., Valli L. (2007). STAT3 is required for IL-6-gp130—dependent activation of hepcidin in vivo. *Gastroenterology*.

[135] Poli M., Asperti M., Ruzzenenti P., Regoni M., Arosio P. (2014). Hepcidin antagonists for potential treatments of disorders with hepcidin excess. *Frontiers in Pharmacology*.

